# Recurrence after implementation of an updated intraoperative protocol for totally extraperitoneal (TEP) inguinal hernia repair in a high-volume clinic - a retrospective cohort study

**DOI:** 10.1007/s10029-025-03327-6

**Published:** 2025-05-15

**Authors:** R.R. Meuzelaar, A.H.W. Schiphorst, J.P.J. Burgmans

**Affiliations:** 1 Department of Surgery, Diakonessenhuis, Utrecht, The Netherlands; 2 Department of Surgery, Erasmus Medical Center, Rotterdam, The Netherlands

**Keywords:** Totally extraperitoneal repair, Recurrence rate, Mesh fixation, Lipoma reduction

## Abstract

**Purpose:**

Although the recurrence rate after inguinal hernia repair is low, it remains an important postoperative outcome. To further reduce this rate, modifiable risk factors should be addressed. This study assessed reoperations for recurrence-like complaints following adjustments to the intraoperative protocol aimed at preventing recurrent inguinal hernias. These adjustments included fixating mesh in large unilateral direct defects and large bilateral hernias, and thoroughly reducing inguinal lipomas.

**Methods:**

Elective totally extraperitoneal (TEP) repairs in adults performed between January 1, 2013, and October 25, 2023, were retrospectively included. The cohort was subsequently divided into two groups based on the timing of their TEP repair: before (pre-implementation) or after (post-implementation) the protocol adjustments. The primary outcome was reoperation for recurrence-like complaints following the initial TEP repair. Secondary outcomes included mesh fixation and lipoma reduction.

**Results:**

A total of 12,878 TEP repairs in 12,507 patients were included (pre-implementation: 5,454; post-implementation: 7,424). Reoperation rate decreased from 0.97% pre-implementation to 0.65% post-implementation (*p* = 0.630). Following protocol adjustments, mesh fixation for unilateral hernias significantly increased from 3.2% to 5.4% (*p* < 0.001), and for bilateral hernias from 9.8% to 16.3% (*p* < 0.001). Lipoma reduction was similar between the groups (unilateral: 32.3–32.6%, *p* = 0.625; bilateral: 36.0–38.1%, *p* = 0.288).

**Conclusion:**

The adjusted intraoperative protocol demonstrated a non-significant declining trend of reoperations for recurrence-like complaints. Lipoma reduction was already well-implemented. While mesh fixation increased, it had no significant effect on the recurrence rate, so careful selection of hernias requiring fixation warrants attention.

## Introduction

Inguinal hernia repair accounts for the majority of elective surgical procedures worldwide [1]. In the Netherlands, approximately 30,000 of these repairs are performed annually [2]. Hence, even small improvements in this field could have a profound positive impact on quality of life. Chronic postoperative inguinal pain (CPIP) and recurrence are the most common complications of inguinal hernia surgery. The number of recurrences has decreased since the introduction of mesh repairs. Consequently, recurrence rates are currently low, with 1.8% for mesh repair and 4.0% for non-mesh repair [3]. However, recurrence remains one of the most important outcomes because it often necessitates reoperation, increasing morbidity and healthcare costs [4, 5]. Furthermore, the exact recurrence rate is difficult to determine, as not all patients undergo surgery for a recurrent hernia, and some may have the operation at a different clinic. Therefore, reoperation is often used as a surrogate for recurrence, which is an underestimation of the true number of recurrences.

Risk factors for recurrence can be classified as either patient- or surgeon-related. Patient-related risk factors consist primarily of non-modifiable factors, such as female sex, direct inguinal hernias at the time of the primary procedure, and surgery for a recurrent inguinal hernia [6]. Surgeon-related risk factors, for instance poor operative techniques, lack of experience, and low surgical volume, are potentially modifiable and can be addressed to reduce recurrence rate [7, 8]. Roos et al. identified possible modifiable causes of recurrence after primary totally extraperitoneal (TEP) repair in a high-volume setting, minimizing the influence of surgeon-related risk factors [9]. First, the authors concluded that mesh fixation should be considered in large unilateral direct defects and large bilateral hernias [9]. “Large” was defined as defects exceeding 3 cm, in accordance with the European Hernia Society’s (EHS) definition [10]. This approach aligns with the recommendations of international guidelines for groin hernia management [7, 11]. Second, thorough inspection of the extraperitoneal space for lipomas and their removal was advised, as the remaining lipomas can mimic the symptoms of an inguinal hernia [12, 13].

Both recommendations were integrated into the intraoperative protocol in approximately September 2017 and fully adopted by January 2018 in our high-volume hernia clinic. The objective of the current study was to assess the impact of these adjustments on reoperation for recurrence-like complaints following initial TEP repair. We hypothesize that these adjustments have led to a reduction in reoperations for recurrence-like complaints, as well as an increase in mesh fixation and lipoma reduction.

## Methods

### Patients

The present study retrospectively examined all inguinal hernia repairs performed at a high-volume specialized hernia clinic between January 1, 2013, and October 25, 2023. The implementation of recommendations by Roos et al. [14] was the only change to the surgical protocol during this period. Approximately 1,400 procedures were performed annually by experienced hernia surgeons who had completed the learning curve (> 500 procedures). Throughout the study period, six hernia surgeons performed repairs at this clinic. Only elective TEP repairs in adult patients were included in the analysis.

The hernia surgeons had regular meetings to review the technical aspects of the procedure and assess potential modifications for implementation. The implementation of the updated intraoperative protocol began with a meeting in September 2017, during which the findings of Roos et al. were discussed. While the timeline for adoption may have varied slightly among surgeons due to individual schedules, all surgeons implemented the changes by January 2018. Subsequently, the cohort was divided into two groups, depending on when their TEP repair had been performed: pre-implementation (January 1, 2013 – December 31, 2017) and post-implementation (January 1, 2018 – October 25, 2023) of the updated intraoperative protocol. Approval of the Institutional Review Board was obtained. The study adhered to the Strengthening the Reporting of Observational Studies in Epidemiology (STROBE) guidelines [14].

## Clinical practice

All patients are operated under general anaesthesia with a standardized surgical technique and a synthetic, nonabsorbable, heavyweight, microporous 10 × 15 cm polypropylene mesh is placed preperitoneally [15]. Surgery is performed as a day-case procedure and patients are discharged the same day unless adverse events prohibit early discharge. Patients are advised to abstain from strenuous activities during the first postoperative week. A telephone follow-up consult is conducted six to eight weeks after surgery. If recurrence-like complaints are suspected, patients either present on their own initiative at the outpatient clinic or are referred by their general practitioner. At the clinic, history taking, physical examination and if necessary, additional imaging (ultrasonography, MRI, or CT) are performed. The decision to reoperate is based on clinical and/or radiological findings, in accordance with the patient’s preferences. In cases of reoperation for recurrence after TEP repair, an anterior open technique (Lichtenstein) is used. A re-TEP is only performed if a recurrence is suspected within one week after the initial TEP repair, referred to as early recurrence. In case of mesh fixation, typically 2–3 tackers are placed along the rectus abdominis in the midline.

## Outcomes

Relevant patient data for each procedure were collected from the electronic patient record, including age, sex, Body Mass Index (BMI), reoperation for recurrence-like complaints, time to recurrence, mortality, and time to death. The primary event for Kaplan-Meier analysis was recurrence defined as reoperation for recurrence-like complaints. Time to death was recorded for patients who died before experiencing a recurrence and these patients were censored at that point. Time to recurrence or death was initially recorded in days and later converted to months. As a surrogate for time to recurrence, the interval from the primary TEP repair until the patient’s first presentation with recurrence-like complaints at the (outpatient) clinic was used.

Intraoperative characteristics were retrieved from the surgical report, containing operative time, side of hernia repair, hernia classification, lipoma reduction, and mesh fixation. If a lipoma was observed, reduced, or removed during unilateral repairs, or on at least one side during bilateral repairs, lipoma reduction was recorded as present. In this clinic, a permanent tacker fixation device was routinely employed as the standard practice, ensuring that all six surgeons used the same fixation method. Mesh fixation was recorded as performed if a fixation device was utilized during TEP repair. Hernia types were classified into the following categories: direct, indirect, and other. The “other” category included femoral and combined hernias for unilateral cases, and femoral, combined, and asymmetrical hernias for bilateral cases. “Asymmetrical” was used for cases involving different hernia types on each side, such as a right-sided direct hernia with a left-sided indirect hernia.

Subgroup analyses were conducted for all reoperated groins. In addition to the aforementioned baseline characteristics, data consisting of recurrence type, time to reoperation, and intra- and postoperative complications were retrieved. The recurrence types were categorized into four groups: true recurrence, new recurrence, isolated lipoma, and no hernia recurrence or lipoma. A recurrence was classified as ‘true’ if the same hernia type was observed during reoperation (e.g., direct-to-direct or indirect-to-indirect). ‘New recurrence’ refers to cases where the hernia type identified during reoperation differed from the type observed during initial TEP repair.

The primary outcome was reoperation for recurrence-like complaints after initial TEP repair. Both procedures, initial TEP repair and reoperation for recurrence-like complaints, had to be performed in our clinic to audit our results. Secondary outcomes were mesh fixation and lipoma reduction during initial TEP repair.

### Statistical analysis

Statistical analyses were performed with R Statistical Software (R v4.3.2; RStudio v2023.09.1; R Core Team). A Kaplan-Meier analysis was utilized to assess the primary outcome for both groups in combination with a log-rank test for between-group analysis. Secondary outcomes were analyzed with Pearson’s Chi-square or Fisher’s exact test. Descriptive statistics were used for baseline characteristics including median and interquartile range (IQR) or mean and standard deviation (SD) for continuous variables and counts with percentages for categorical variables. Between-group comparisons were performed with the Student’s t-test or Wilcoxon rank-sum test. Data which were not registered in the electronic patient record were considered missing data.

## Results

**Fig. 1:**
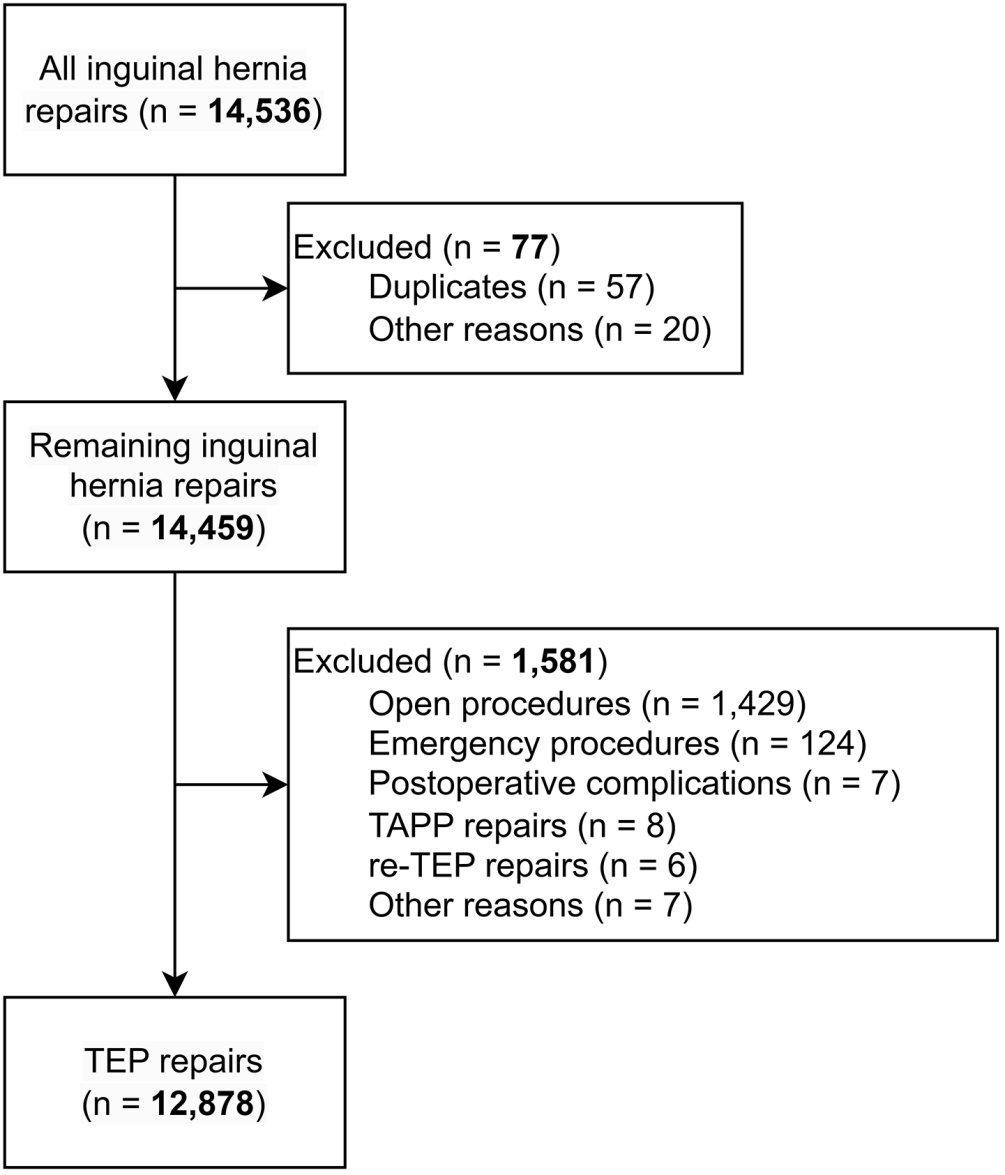
Flowchart of inclusions. TAPP, transabdominal preperitoneal; TEP, totally extraperitoneal

During the study period, 14,536 inguinal hernia repairs were performed. Duplicates were identified and excluded from the analysis. Of these procedures, 12,878 TEP repairs in 12,507 patients were included (pre-implementation *n* = 5,454; post-implementation *n* = 7,424). Figure 1 displays the reasons for exclusion. Significantly more male patients (93.6%) were treated after the adoption of the intraoperative protocol (*p* = 0.017) compared to the pre-implementation group (92.5%). Patients treated after protocol adoption were also significantly older (median 60.9 years (IQR: 49.6–70.1)) than those treated before protocol implementation (median 58.2 years (IQR: 46.5–67.5)) (*p* < 0.001). Table 1 summarizes the baseline characteristics of both groups.

**Table 1:** Baseline characteristics per procedure

	Pre-implementation (*n* = 5,454)	Post-implementation (*n* = 7,424)	*p*-value
Sex, n (%)			**0.015**
Male	5,043 (92.5)	6,946 (93.6)	
Female	411 (7.5)	478 (6.4)	
BMI, mean (SD), kg/m^2^	24.8 (3.0)	24.9 (3.0)	0.119
Age, median (IQR), years	58.2 (46.5–67.5)	60.9 (49.6–70.1)	**< 0.001**
Location of hernia, n (%)			0.460
Unilateral	4,577 (83.9)	6,194 (83.4)	
Bilateral	877 (16.1)	1,230 (16.6)	

BMI, body mass index; SD, standard deviation; IQR, interquartile range

### Reoperation

**Fig. 2:**
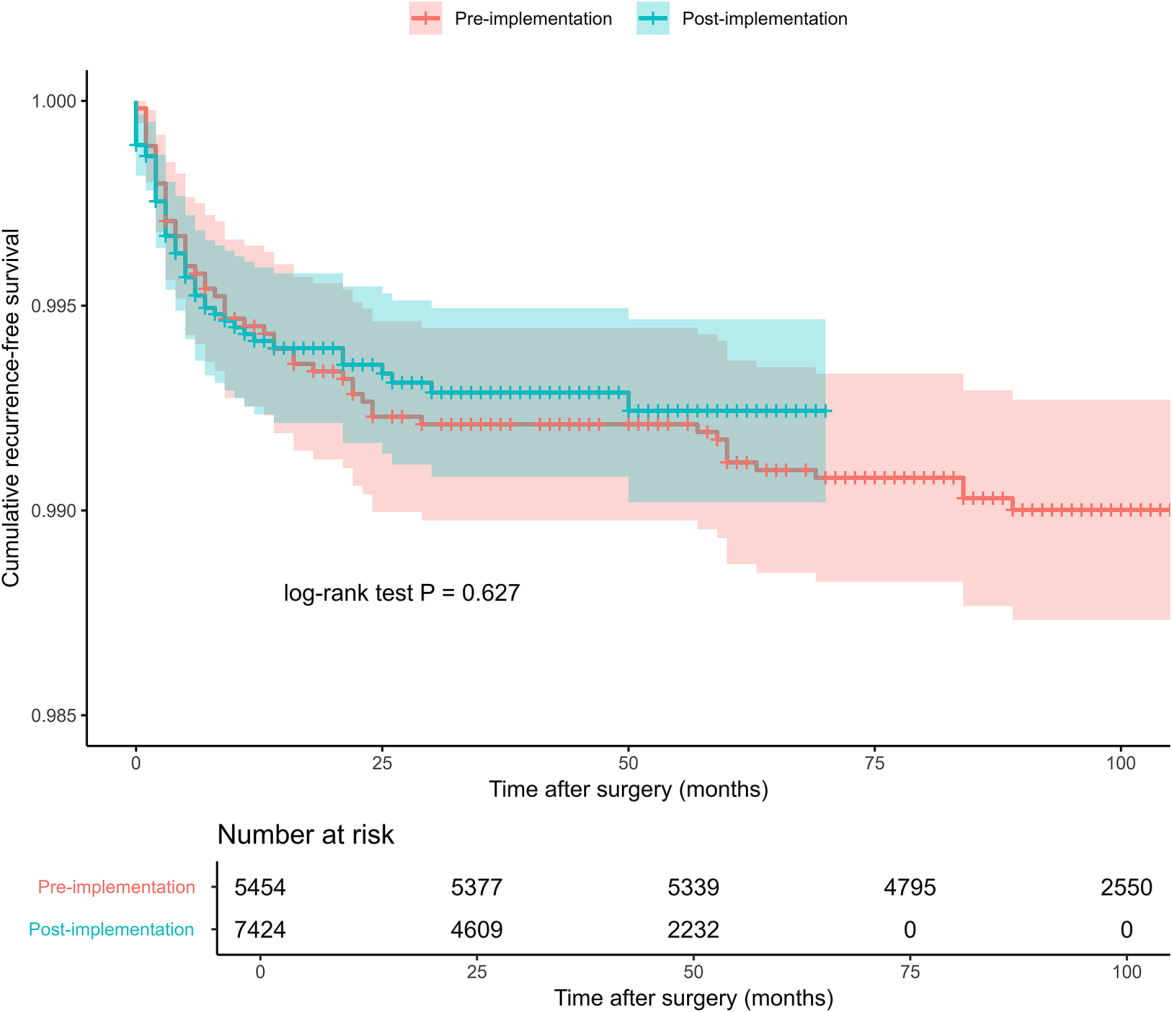
Kaplan-Meier survival curve with recurrence-free survival estimates and risk table

Prior to adoption of the updated intraoperative protocol, reoperation for recurrence-like complaints was observed in 53 out of 5,454 procedures (0.97%). In the post-implementation group, reoperations occurred in 48 out of 7,424 procedures (0.65%). Figure 2 presents the cumulative recurrence-free survival of both groups. The log-rank test yielded *p* = 0.630, which was not statistically significant. The median time to recurrence in the pre-implementation group was 9.0 months (IQR: 3.0–23.0), whereas this was 3.5 months (IQR: 2.0–7.3) in the post-implementation group.

## Secondary outcomes

### Mesh fixation and lipoma reduction for unilateral hernias

Lipoma reduction was similar between the pre- and post-implementation groups for unilateral TEP repairs (32.3% pre-implementation vs. 32.6% post-implementation; *p* = 0.625). It is worth noting that the median operative time significantly decreased from a median of 17 minutes (IQR: 14–22) to 15 minutes (IQR: 12–20) after protocol adjustments (*p* < 0.001). Furthermore, the use of fixation devices significantly increased from 3.2% to 5.4% after 2018 (*p* < 0.001). Specifically, mesh fixation was performed more frequently in both direct (2.3% pre-implementation vs. 3.6% post-implementation) and indirect (0.5% vs. 1.2%) hernias (*p* < 0.001). All operative characteristics are outlined in Table 2.

**Table 2:** Operative characteristics for unilateral procedures

	Pre-implementation (*n* = 4,577)	Post-implementation (*n* = 6,194)	*p*-value
Hernia location, n (%)			0.076
Left	1,944 (42.5)	2,737 (44.2)	
Right	2,633 (57.5)	3,457 (55.8)	
Hernia type, n (%)			**0.022**
Direct	1,051 (23.0)	1,278 (20.6)	
Indirect	3,215 (70.2)	4,439 (71.7)	
Other	310 (6.8)	440 (7.1)	
Missing	1 (0.0)	37 (0.6)	
Lipoma reduction, n (%)	1,479 (32.3)	2,022 (32.6)	0.625
Operative time, median (IQR), minutes	17.0 (14.0–22.0)	15.0 (12.0–20.0)	**< 0.001**
Mesh fixation, n (%)	148 (3.2)	336 (5.4)	**< 0.001**
Direct	103 (2.3)	224 (3.6)	**< 0.001**
Indirect	24 (0.5)	75 (1.2)	**< 0.001**
Other	21 (0.4)	37 (0.6)	0.321

IQR, interquartile range

### Mesh fixation and lipoma reduction for bilateral hernias

Although more lipomas were reduced in bilateral repairs post-implementation (38.1%), this difference was not statistically significant (pre-implementation: 36.0%; *p* = 0.288). Additionally, the operative time for bilateral repairs significantly decreased post-implementation from a median of 25 minutes (IQR: 21–32) to 21 minutes (IQR: 17–27) (*p* < 0.001). Following the adoption of the updated intraoperative protocol, mesh fixation was performed significantly more frequently in both direct hernias (5.5% pre-implementation vs. 10.1% post-implementation; *p* < 0.001) and other hernias (2.3% vs. 4.4%; *p* = 0.007). Table 3 provides all operative characteristics for bilateral procedures.

**Table 3:** Operative characteristics for bilateral procedures

	Pre-implementation (*n* = 877)	Post-implementation (*n* = 1,230)	*p*-value
Hernia type, n (%)			**0.018**
Direct	340 (38.8)	435 (35.4)	
Indirect	375 (42.8)	500 (40.7)	
Other	162 (18.4)	288 (23.4)	
Missing	0 (0.0)	7 (0.5)	
Lipoma reduction, n (%)	316 (36.0)	469 (38.1)	0.288
Operative time, median (IQR), minutes	25.0 (21.0–32.0)	21.0 (17.0–27.0)	**< 0.001**
Mesh fixation, n (%)	86 (9.8)	201 (16.3)	**< 0.001**
Direct	48 (5.5)	124 (10.1)	**< 0.001**
Indirect	18 (2.0)	22 (1.8)	0.675
Other^a^	20 (2.3)	55 (4.4)	**0.007**

IQR, interquartile range

^a^ Over 80% of patients had at least a direct hernia on one side

### Recurrence subgroup analysis

Overall, 102 reoperations for recurrent symptoms were performed in 101 patients (one patient was reoperated bilaterally after initial bilateral TEP repair). Baseline characteristics were comparable between the groups (Table 4). Significantly fewer lipomas were observed during reoperation after implementation of protocol adjustments (18.5% pre-implementation vs. 6.3% post-implementation; *p* = 0.041). Additionally, a significantly higher number of recurrences occurred within the first week in the post-implementation group (0% pre-implementation vs. 12.5% post-implementation; *p* = 0.041). Among these six patients, two presented with a major scrotal hernia during the preoperative physical examination. In two others, the surgical report noted a large direct hernia intraoperatively, requiring mesh fixation. Half of these early recurrences involved an initial bilateral repair. Only one case of intraoperative bleeding was observed in the pre-implementation group. The following postoperative complications were seen before the implementation of the updated intraoperative protocol: superficial wound infection (*n* = 2), epididymitis (*n* = 1), and syncope with unknown cause (*n* = 1). Post-implementation postoperative complications included: urinary retention (*n* = 2), hematoma (*n* = 1), and ileus due to recurrence of the hernia causing incarceration. The amount of intraoperative and postoperative complications was comparable between the groups (*p* = 1.0).

**Table 4:** Characteristics of reoperated groins (*n* = 102)

	Pre-implementation (*n* = 54)	Post-implementation (*n* = 48)	*p*-value
Sex, n (%)			1.00
Male	52 (96.3)	47 (97.9)	
Female	2 (3.7)	1 (2.1)	
BMI, mean (SD), kg/m^2^	25.9 (3.2)	26.4 (3.3)	0.488
Age at primary TEP repair, median (IQR), years	62.6 (50.7–67.9)	65.4 (54.5–71.6)	0.102
Primary hernia location, n (%)			0.838
Unilateral	36 (66.7)	31 (64.6)	
Bilateral	18 (33.3)	17 (35.4)	
Recurrence type, n (%)			**0.041**
True recurrence	32 (59.3)	35 (72.9)	
New recurrence	12 (22.2)	7 (14.5)	
Isolated lipoma	10 (18.5)	3 (6.3)	
No hernia recurrence or lipoma	0 (0.0)	3 (6.3)	
Time to reoperation, n (%)			**0.020**
≤ 1 week	0 (0.0)	6 (12.5)	
≤ 30 days	4 (7.4)	3 (6.2)	
31–90 days	8 (14.8)	12 (25.0)	
> 90 days	42 (77.8)	27 (56.3)	

BMI, body mass index; SD, standard deviation; IQR, interquartile range;

## Discussion

This retrospective cohort study demonstrated that adopting the updated intraoperative protocol, which included mesh fixation for large unilateral direct defects and large bilateral hernias, along with thorough inspection and removal of lipomas in the extraperitoneal space, led to a non-significant declining trend (*p* = 0.630) in reoperations for recurrence-like complaints. Mesh fixation during initial unilateral and bilateral TEP repairs significantly increased post-implementation (*p* < 0.001), particularly in direct and indirect unilateral hernias, as well as direct and other bilateral hernias. Lipoma reduction remained comparable between the groups (unilateral: 32.3–32.6%, *p* = 0.625; bilateral: 36.0–38.1%, *p* = 0.288).

Recurrence risk can be influenced by surgeon-related factors such as surgical technique and experience. International guidelines recommend laparoendoscopic techniques in unilateral hernias because they produce faster recovery and less pain than an open mesh (Lichtenstein) approach [16, 17]. Nonetheless, the learning curve for laparoendoscopic repair, particularly the TEP technique, is longer, making sufficient experience crucial for achieving favourable outcomes [17, 18]. In fact, surgeon expertise will invariably affect the results of any surgical technique. This point is illustrated by the revisions of the updated Herniasurge International Guide­lines, which have revised their recommendations regarding non-mesh repair based on two high-quality database studies [19, 20]. These two studies demonstrated that the Shouldice technique, if expertise was present, achieved similar 1-year outcomes to Lichtenstein and laparoendoscopic procedures in selected patients [19, 20].

Furthermore, Malik et al. concluded that hospital specialization affects the risk of hernia recurrence after inguinal hernia repair. The results of their study showed that non-mesh repair at the Shouldice Hospital, a high-volume specialized clinic, was associated with a significantly lower risk of reoperation for recurrence than similar repairs at local general hospitals [21]. These findings suggest that high-volume, experienced surgeons achieve better outcomes than their less specialized counterparts, regardless of the technique or patient population. This fact is further underscored by the success of other high-volume hernia clinics, which consistently achieve lower recurrence rates [8, 22, 23]. In these clinics, protocol changes can be evaluated quickly through Plan-Do-Check-Act (PDCA) cycles, as substantial data is generated in a short period and the influence of surgeon-related risk factors on postoperative outcomes is minimized. PDCA cycles ensure internal quality control by promoting structured, data-driven improvements that enhance postoperative outcomes and shed light on factors like recurrence patterns. In the current practice, the technique is standardized, the surgeon’s experience exceeds the TEP repair learning curve (> 500 repairs), and all procedures are performed in a high-volume setting. Considering these surgeon-related factors helps maintain low recurrence rates.

In both unilateral and bilateral defects, mesh fixation significantly increased following the implementation of the updated intraoperative protocol, especially in direct defects. Notably, mesh fixation was performed significantly more frequently in unilateral indirect hernias (0.5% pre-implementation vs. 1.2% post-implementation; *p* < 0.001). According to the Herniasurge Guidelines, mesh fixation is strongly recommended only for large direct hernias (type M3, EHS classification), based on expert opinion [7]. Whereas, the International EndoHernia Society (IEHS) Guidelines recommend mesh fixation for both large direct and indirect hernias (EHS: M3, L3). For large indirect defects (> 4–5 cm), they advise using non-traumatic fixation (e.g., glue) to the psoas muscle, instead of the fixation device with tackers used in the current study [11]. Our findings suggest that mesh fixation may have been overused, with a significant increase in unilateral indirect hernias but no difference in recurrence rate. Additionally, the cost of fixation devices must be considered. Fixation carries the potential risk of CPIP; however, Gutlic et al. demonstrated that for primary inguinal hernia repairs in men the amount of patients with CPIP were comparable between mesh fixation and non-fixation in a nationwide population-based study [24]. Multiple systematic reviews have also demonstrated that there is no significant difference in postoperative pain between permanent tack fixation and non-fixation in either TEP or transabdominal preperitoneal (TAPP) procedures [25–27]. In addition, the exact size of the hernia defect (in centimeters) was not documented in the surgical reports of this cohort. If a defect larger than 3 cm was visually observed, the surgeon would document it in the surgical report as ‘big’ or ‘large’; however, this was not done routinely. This lack of consistent documentation limits our ability to determine whether mesh fixation was justified in these cases, representing a notable limitation of the study.

Lipoma reduction was carried out in 32–38% of the repairs and remained consistent over time, likely because it was already thoroughly performed prior to implementation. A cord lipoma is defined as isolated fatty tissue originating from the retroperitoneal tissue and bulging through the internal ring, similar to an indirect hernia [12]. In males, this lipoma can be easily separated from the cord structures and is distinct from fatty tissue accompanying the testicular vessels [28]. Dias et al. reported a notably high incidence of 65% for cord lipomas during laparoscopic or robotic inguinal hernia repair (*n* = 141) [29]. This incidence may represent an overestimation, as other studies have reported an overall incidence of 21% to 27%, which is more in line with our findings [12, 13, 30]. One additional study indicated a high incidence of 72%, but this was limited to indirect hernia types only [28]. For this reason, it is important to make a distinction between fat from the spermatic cord, which should be left alone as relentless manipulation may cause injury to the blood supply of the testis and a true cord lipoma, which originates from preperitoneal fat [30]. In a survey on the management of cord lipomas, only 7% of the surgeons (*n* = 58) answered that a lipoma should be left untouched [31]. Additionally, 30% of respondents stated they would not document this finding in the surgical report [31]. Resection of spermatic cord or round ligament lipomas remains important to prevent future symptoms; therefore, their removal should be well-documented.

In the subgroup analysis of hernia recurrence types, the number of lipomas identified during reoperation significantly decreased, leading to a relative increase in true recurrences (*p* = 0.041). The implemented intraoperative protocol adjustments appear to be achieving the desired effect, as Roos et al. reported that in 18% of their reoperated patients an isolated lipoma was found, which has been further reduced to 6% in our study [9]. Another notable finding is that six additional patients underwent reoperation within one week in the post-implementation group (*p* = 0.020). The literature’s definition of early recurrence is indefinite and may vary from less than one week to up to two years postoperatively [32]. Immediate postoperative recurrences are generally related to technical failure, but may also arise from undetected hernias or lipomas during the initial repair, profound increases in intra-abdominal pressure, or direct trauma [32]. As recurrence rates remain low in both groups, the resulting subgroups are relatively small. Additionally, the absence of a standardized definition for early recurrence leads to arbitrary cut-off points, warranting cautious interpretation of this result. A possible explanation for the significant increase of recurrences within one week postoperatively may be attributed to changes in our clinical approach. The indications for performing TEP repair in our clinic have been expanded, allowing large scrotal hernias to be treated endoscopically, cases that might previously have been managed with a Lichtenstein repair. Also, surgeons used to be more cautious and conservative regarding immediate postoperative groin swelling, whereas re-TEP performed within one week is now considered safe and is becoming more common.

A major strength of this cohort is the large patient sample size. In this study, the recurrence rate was low in both groups (pre-implementation: 0.97%; post-implementation: 0.65%). Despite the large size of both groups, no statistically significant difference in recurrence rate was observed. In retrospect, a sample size calculation was performed, which determined that 12,315 patients per arm were required (alpha = 0.05, beta = 0.2, power = 0.80) to detect a significant effect. Given the low recurrence rates, the post-implementation group would need to be nearly twice as big to achieve sufficient statistical power, requiring at least four more years of follow-up, which is a limitation of the study. When surgeon-related factors influencing recurrence rates are minimized as much as possible, other areas for improvement may still remain. However, reducing recurrence rates to near zero has its limits, as non-modifiable patient-related risk factors may still persist.

## Conclusions

So far, a declining non-significant trend of reoperations for recurrence-like complaints after initial TEP repair was observed after the updated intraoperative protocol was implemented. Lipoma reduction was already consistently performed, but it may still help reduce recurrence rates in other hospitals. Although mesh fixation increased, it did not significantly affect recurrence rate, highlighting the need for careful selection of cases that require fixation. To further optimize surgical outcomes, modifiable factors should be systematically analyzed and refined using PDCA cycles for internal quality control.
