# Correction to: Recurrence after implementation of an updated intraoperative protocol for totally extraperitoneal (TEP) inguinal hernia repair in a high-volume clinic - a retrospective cohort study

**DOI:** 10.1007/s10029-025-03382-z

**Published:** 2025-06-17

**Authors:** R.R. Meuzelaar, A.H.W. Schiphorst, J.P.J. Burgmans

**Affiliations:** 1 Department of Surgery, Diakonessenhuis, Utrecht, The Netherlands; 2 Department of Surgery, Erasmus Medical Center, Rotterdam, The Netherlands

**Correction to: Hernia (2025) 29:164**

10.1007/s10029-025-03327-6

Several errors have been introduced in the original article.

The original version has now been revised.

In this article, Fig. 1 appeared as.

**Fig. 1:**
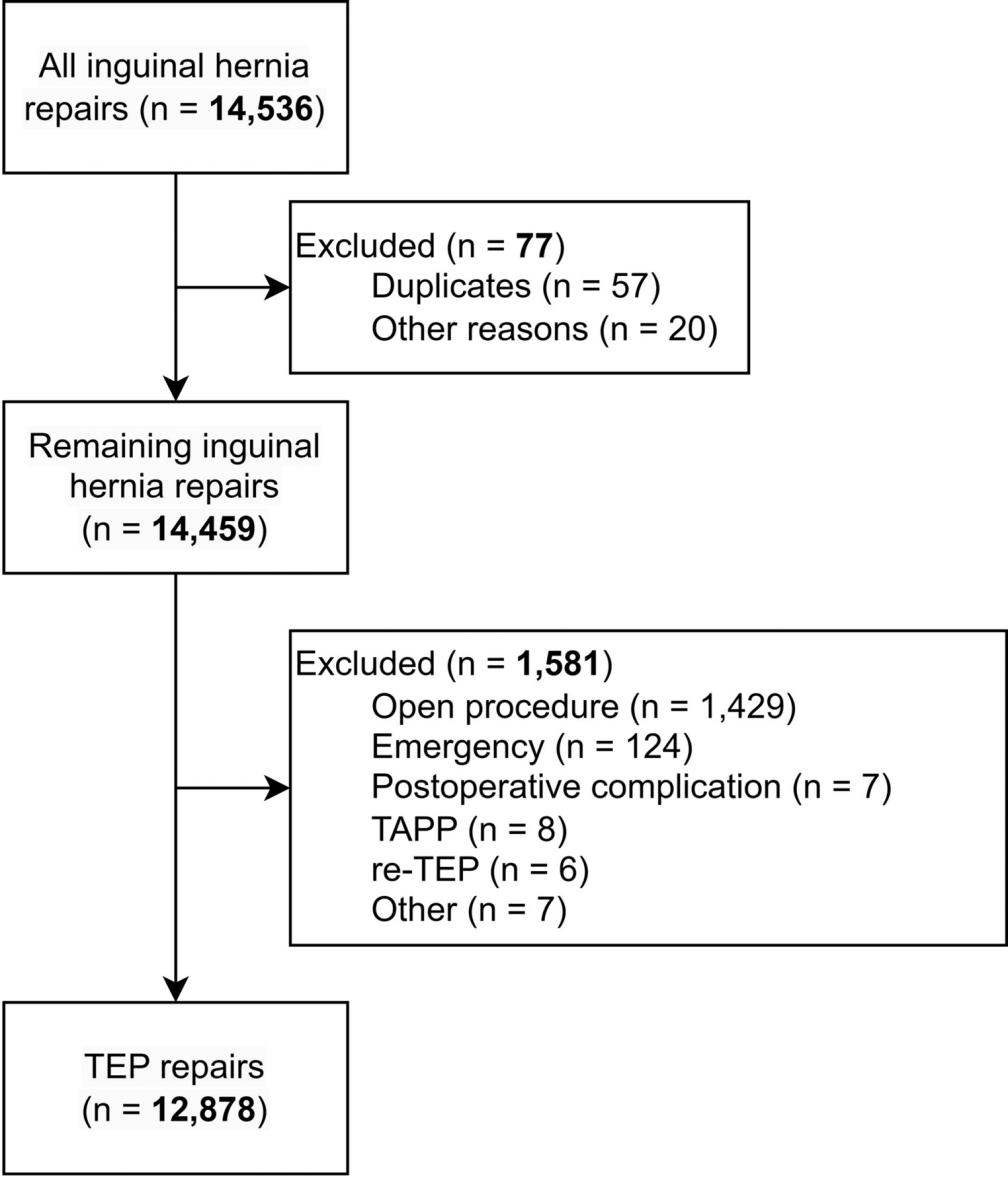
Flowchart of inclusions

but should have appeared as.

**Fig. 1:**
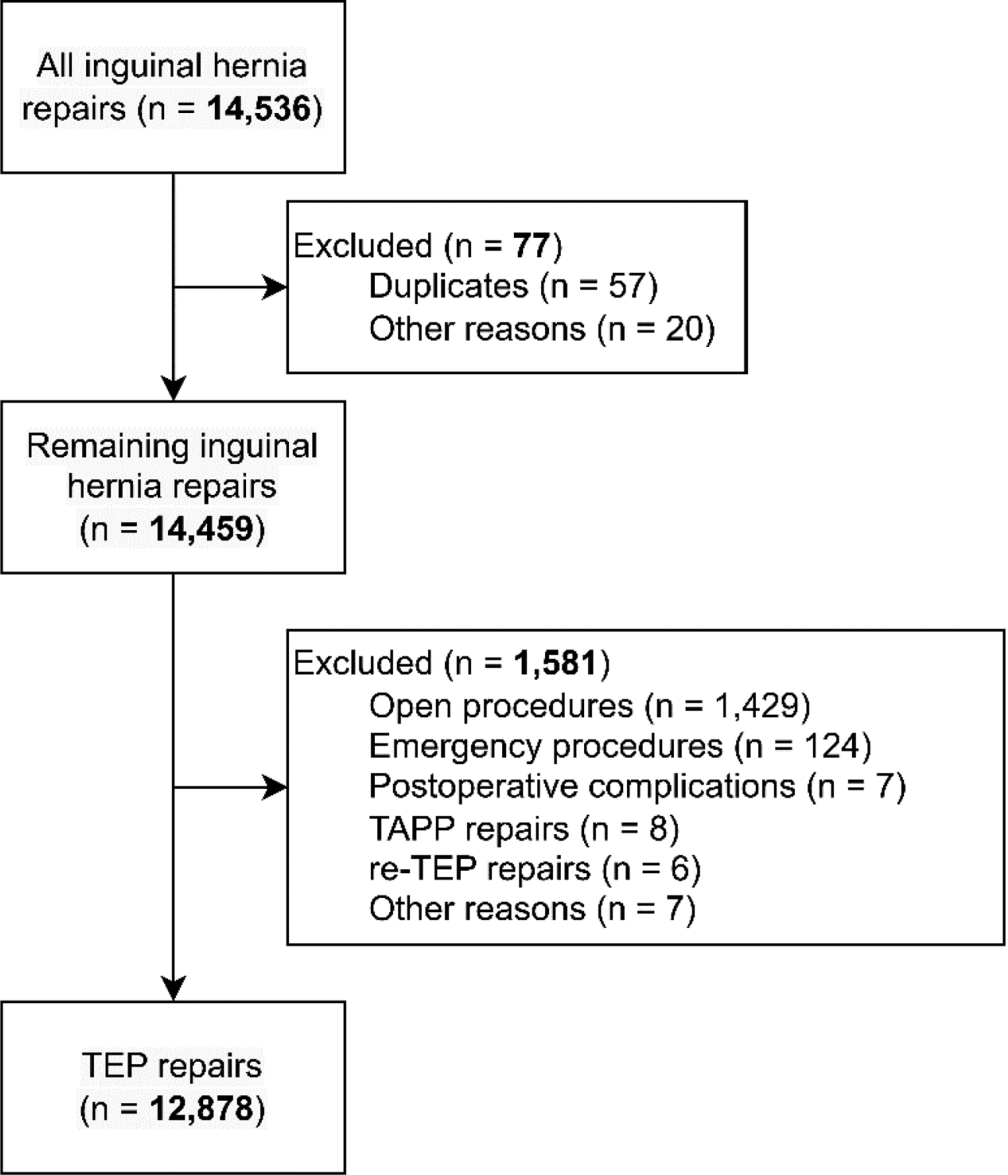
Flowchart of inclusions

The publisher apologize for this mistake.

The original article has been corrected.

